# Vitamin D supplementation to the older adult population in Germany has the cost‐saving potential of preventing almost 30 000 cancer deaths per year

**DOI:** 10.1002/1878-0261.12924

**Published:** 2021-03-10

**Authors:** Tobias Niedermaier, Thomas Gredner, Sabine Kuznia, Ben Schöttker, Ute Mons, Hermann Brenner

**Affiliations:** ^1^ Division of Clinical Epidemiology and Aging Research German Cancer Research Center (DKFZ) Heidelberg Germany; ^2^ Medical Faculty Heidelberg University of Heidelberg Germany; ^3^ Network Aging Research (NAR) University of Heidelberg Germany; ^4^ Cancer Prevention Unit German Cancer Research Center (DKFZ) Heidelberg Germany; ^5^ Division of Preventive Oncology German Cancer Research Center (DKFZ) and National Center for Tumor Diseases (NCT) Heidelberg Germany; ^6^ German Cancer Consortium (DKTK) German Cancer Research Center (DKFZ) Heidelberg Germany

**Keywords:** cancer mortality, cost saving, prevention, supplementation, vitamin D

## Abstract

Recent meta‐analyses of randomized controlled trials (RCTs) have demonstrated significant reduction in cancer mortality by vitamin D supplementation. We estimated costs and savings for preventing cancer deaths by vitamin D supplementation of the population aged 50+ years in Germany. Our analysis is based on national data on cancer mortality in 2016. The number of preventable cancer deaths was estimated by multiplying cancer deaths above age 50 with the estimated proportionate reduction in cancer mortality derived by vitamin D supplementation according to meta‐analyses of RCTs (13%). Saved costs were estimated by multiplying this number by estimated end‐of‐life cancer care costs (€40 000). Annual costs of vitamin D supplementation were estimated at 25€ per person above age 50. Comprehensive sensitivity analyses were conducted. In the main analysis, vitamin D supplementation was estimated to prevent almost 30 000 cancer deaths per year at approximate costs of €900 million and savings of €1.154 billion, suggesting net savings of €254 million. Our results support promotion of supplementation of vitamin D among older adults as a cost‐saving approach to substantially reduce cancer mortality.

## Graphical abstract

Vitamin D supplementation to Germany's population aged 50 years and above was estimated to prevent almost 30 000 cancer deaths per year at approximate costs of €900 million and savings of €1.154 billion. Thus, supplementation of vitamin D among older adults appears to be a cost‐saving approach for reducing cancer mortality.

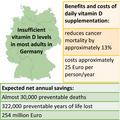

**Abbreviations**
25(OH)D: 25‐hydroxyvitamin D
CI: confidence interval
HER2: human epidermal growth factor receptor 2
HR: hormone receptor
IQR: interquartile range
IU: international units
RR: relative risk
SD: standard deviation
YLL: years of life lost

## 1 Introduction

Approximately 9.6 million people died from cancer in 2018 globally [1], and this number is expected to increase to 16.4 million by 2040. Corresponding numbers for Germany are 247 000 and 326 000 [1], respectively, denoting a relative increase by 32%.

Undoubted progress in cancer therapy, and for some cancers, also in early detection, has contributed to a steady slow decline in age‐adjusted cancer mortality in many countries in the past decades. Between 2007 and 2017, age‐adjusted cancer mortality decreased in 145 out of 195 countries, despite an increase in age‐standardized incidence in the majority (*n* = 123) of countries [2].

Reduction in mortality by progress in therapy comes at high costs, though. For example, 16 drugs were approved for the treatment of advanced or metastatic gastrointestinal cancers by the US Food and Drug Administration between 2006 and 2017 [3]. Median total drug price over the median treatment duration per patient was $62 415. In 28 supporting clinical trials, the median benefit of overall survival was 1.9 months.

Randomized clinical trials (RCTs) on vitamin D supplementation have rather consistently shown a decrease in cancer mortality. Although this association failed to reach statistical significance in single trials, a recent meta‐analysis of five RCTs published until November 2018 yielded a significant reduction in total cancer mortality by 13% (95% confidence interval, CI: 4–21%) [4]. Given the low costs of vitamin D supplementation, it could also be highly cost‐effective.

In this study, we estimated savings and costs of reducing cancer mortality by vitamin D supplementation in Germany.

## 2 Methods

### 2.1 Data sources

Numbers of deaths and years of life lost (YLL) due to cancer in Germany were estimated for 2016, the most recent year for which cancer mortality data were available at the time of analysis [5]. Sex‐ and age‐specific numbers of cancer deaths were obtained from the German Center for Cancer Registry Data. Further life expectancy at the age of cancer deaths was obtained from the latest available life table (2016/2018) of the Federal Statistical Office of Germany. We focused our analysis on the population aged 50+, given that the vast majority of cancer deaths occur above age 50 (> 96% of cancer deaths in Germany [5]) and participants in vitamin D trials were mostly > 50 years old. Numbers of deaths and further life expectancy are shown by age and sex in Table S1.

Estimates of effects of vitamin D supplementation on cancer mortality from the three most recent meta‐analyses of RCTs [4, 6, 7], all published in 2019, and their main characteristics, are summarized in Table 1. Another meta‐analysis that was also published in 2019 is not listed because it included studies on cancer incidence in the meta‐analysis on cancer mortality [8]. The summary estimate for cancer mortality reduction was 13% in all three meta‐analyses without this error. For our analysis, we selected the estimate of 13% (95% CI: 4–21%) from the meta‐analysis of Keum *et al*. [4], which included studies with at least 1 year of follow‐up (actual range: 3–7 years). Characteristics of the five RCTs included in that meta‐analysis [9, 10, 11, 12, 13] are summarized in Table 2. Reduction in cancer mortality by ≥ 11% was found in all studies with daily supplementation, whereas essentially no effect was seen in one out of two studies with supplementation by an initial large bolus, followed by monthly bolus supplementations. Thus, we focused on regular daily vitamin D3 supplementation rather than vitamin D3 supplementation by an initial large bolus. Daily vitamin D3 supplementation in the trials reporting on total cancer mortality included in the meta‐analysis by Keum *et al*. ranged from 400 to 2000 international units (IU). For our main analysis, we assumed that daily supplementation of 1000 IU per day will result in a cancer mortality reduction by 13%. In sensitivity analyses, the cancer mortality reduction was varied between 4% and 21%, reflecting the upper and lower end of the 95% CI of the summary effect reported by Keum *et al*. [4]. In further sensitivity analyses, assumed supplementation was varied between 400 and 2000 IU per day, the lower and upper end of the range of doses provided in the trials, and we assumed cancer mortality reductions of 11% and 17% as observed in the respective trials. We used price data from the ‘Red List’ (German: Rote Liste), a directory of medications marketed in Germany [14] from which drug costs of approximately 10, 25, and 50€ per year can be derived for supplementation with 400, 1000, and 2000 IU per day, respectively. In agreement with the RCTs, we assumed supplementation of the entire population aged ≥ 50 without prior laboratory measurement of vitamin D status and hence did not consider costs for such laboratory analyses.

**Table 1:** Recent meta‐analyses of RCTs of vitamin D supplementation and cancer mortality. NR, not reported.

First author, year, reference	Databases searched	Literature searched until	Number of included studies (references)	Included participants	Cancer deaths	Statistical model for pooling	RR (95% CI)
Keum 2019 [4]	PubMed, EMBASE	November 2018	5 [9, 10, 11, 12, 13]	75 241	1107	Random effects	0.87 (0.79–0.96)
Haykal 2019 [6]	PubMed, EMBASE, CENTRAL	December 2018	5 [9, 11, 13, 39, 40]	31 163	1533	Random effects	0.87 (0.79–0.96)
Zhang X 2019 [7]	PubMed, EMBASE	August 2018	7 [9, 10, 11, 12, 13, 41, 42]	NR	1763	Random effects	0.87 (0.79–0.95)

**Table 2:** Characteristics of the five studies included in the meta‐analysis of vitamin D supplementation and cancer mortality from Keum *et al*. [4]. 25(OH)D, 25‐hydroxyvitamin D; IQR, interquartile range; SD, standard deviation.

First author, year (reference)	Country	Participants	% Women	Mean age (age range) [years]	Baseline 25(OH)D [nmol·L^−1^]	Supplementation dose	Duration of intervention [years]	Follow‐up [years]	RR (95% CI) for cancer mortality
Trivedi 2003 [9]	UK	*N* = 2686; doctors	31.9	74.8 (65–85)	Not measured	100 000 IU/4 months	5	5	0.86 (0.61–1.20)
Wactawski‐Wende 2006 [10]	USA	*N* = 36 282; post‐menopausal women	100	50–79	Median (IQR) 42.4 (31.0–58.3)	400 IU per day	Mean 7	Mean 7	0.89 (0.77–1.03)
Avenell 2012 [11]	UK	*N* = 5292; previous low‐trauma fracture	84.7	77 (≥ 70)	Mean 38	800 IU per day	2–5	3	0.85 (0.68–1.06)
Scragg 2018 [12]	New Zealand	*N* = 5110; residents of Auckland	41.9	65.9 (50–84)	Mean (SD) 66.3 (22.5)	200 000 IU initial bolus + 100 000 IU per month	Median (range) 3.3 (2.5–4.2)	Median 3.3	0.99 (0.60–1.64)
Manson 2019 [13]	USA	*N* = 25 871 71% white, 20.2% black, 4% Hispanic	50.6	67.1 (men ≥ 50, women ≥ 55)	Median 71	2000 IU per day	3–6	Median (range) 5.3 (3.8–6.1)	0.83 (0.67–1.02)

End‐of‐life cancer care causes substantial costs, which are much higher than end‐of‐life care costs for other causes of death [15]. Thus, we also compared estimated costs of vitamin D supplementation per prevented YLL to costs for cancer treatment before death in different countries. Data for end‐of‐life cancer treatment costs were taken from published studies on average costs for treatment of all cancers or selected common cancers. Those data are shown in Table S2. For our main analysis, we assumed average end‐of‐life cancer care costs of 40 000€ per person, and we varied this estimate to 20 000€ and 60 000€ in sensitivity analyses.

### 2.2 Statistical analysis

Annual costs of vitamin D supplementation were compared to savings of end‐of‐life cancer care costs. In sensitivity analyses in which costs exceeded savings, we calculated the excess costs per prevented YLL. Given the uncertainties in monetary savings from prevented cancer deaths, we additionally carried out analyses quantifying costs per prevented YLL not considering any such savings and exclusively focusing on costs of supplementation per prevented YLL.

No patients were involved in this modeling study, and thus, no ethics approval was required. Our model comprised absolute numbers of cancer deaths in Germany in 2016 in 5‐year age groups, stratified by sex, further life expectancies at midpoints of age intervals, annual costs of vitamin D supplementation, and age‐ and sex‐specific size of the German population in 2016. YLLs were calculated as number of cancer deaths at each age group ≥ 50 years multiplied by further life expectancy at the midpoint of each age interval (e.g., age 52 for the age group 50–54). YLLs were summed across sexes and 5‐year age groups. Preventable YLLs were calculated as the product of total YLL with the estimated relative risk (RR) reduction achievable by vitamin D supplementation (13% in our main analysis). All analyses were done in r version 3.6.3 [16].

## 3 Results

Table 3 shows the estimates of total costs and savings, as well as the numbers of prevented cancer deaths and prevented YLL in the German population aged 50 years and older, which included 16.8 million men and 19.2 million women in 2016. Under the assumptions of our main analysis (base case scenario), 28 842 cancer deaths, corresponding to 321 671 YLL, could have been prevented. Total costs of supplementation would amount to 900 million €. Assuming saved costs of 40 000 € per prevented cancer death would translate into approximately 1.154 billion € of saved cancer treatment costs, suggesting that vitamin D supplementation would save 254 million €. Key findings of the base case scenario are summarized in the Fig. S1.

**Table 3:** Estimates of prevented years of life lost (YLL) and associated costs and savings in the German population aged 50 years and older using different assumptions regarding vitamin D supplementation effects, doses, and savings of end‐of‐life cancer care costs. IU, international units; YLL, years of life lost.

Scenario	Supplementation costs/person	End‐of‐life cancer care costs/cancer death	Cancer mortality reduction^a^	Total costs per year (million €)	Total savings per year (million €)	Total net costs (million €)	Prevented cancer deaths	Prevented YLL	€/prevented YLL
Base case	25€ per year	40 000€	13%	900	1154	−254	28 842	321 671	Cost saving
Stronger effect^b^	25€ per year	40 000€	21%	900	1864	−964	46 591	519 623	Cost saving
Weaker effect^c^	25€ per year	40 000€	4%	900	355	+545	8874	98 976	5506
400 IU per day^d^	10€ per year	40 000€	11%	360	976	−616	24 405	272 184	Cost saving
2000 IU per day^e^	50€ per year	40 000€	17%	1800	1509	+291	37 716	420 647	692
Lower end‐of‐life cancer care costs	25€ per year	20 000€	13%	900	577	+323	28 842	321 671	1004
Higher end‐of‐life cancer care costs	25€ per year	60 000€	13%	900	1731	−831	28 842	321 671	Cost saving

^a^ Observed mortality reduction found in individual trials (scenario ‘400 IU per day’, ‘2000 IU per day’) or in the meta‐analysis of Keum *et al*. (all other scenarios).

^b^ Lower bound of 95% CI of effect size for cancer mortality by vitamin D supplementation (RR = 0.79) instead of 0.87.

^c^ Upper bound of 95% CI of effect size for cancer mortality by vitamin D supplementation (RR = 0.96) instead of 0.87.

^d^ Assuming that this lower daily dose of vitamin D would result in a slightly smaller cancer mortality reduction of 11%, as observed in the study by Wactawski‐Wende *et al*. ([10], see Table 2)

^e^ Assuming that this higher daily dose of vitamin D would result in a stronger cancer mortality reduction by 17%, as observed in the study by Manson *et al*. ([13], see Table 2).

In sensitivity analyses assuming stronger and weaker effects of supplementation corresponding to lower and upper confidence limits of the RR estimates of the meta‐analysis, estimates of prevented cancer deaths ranged from 8874 to 46 591 and prevented YLL ranged from 98 976 to 519 623. Again assuming €40 000 saved costs per prevented cancer death, but with a stronger assumed mortality reduction (21%), savings would exceed costs by 964 million €. A smaller mortality reduction (4%) would translate to costs per prevented YLL of €5506. Supplementation of 400 IU per day for €10 per year, assumed to result in 11% mortality reduction, was indicated to be cost‐saving, with savings (976 million €) exceeding total costs (360 million €) by 616 million €.

In further sensitivity analyses, we assumed 13% mortality reduction and supplementation costs of 25€ per person (just like in the base case scenario), but varied end‐of‐life cancer care costs to 20 000 and 60 000 €. While the lower end‐of‐life costs would correspond to costs per prevented YLL of approximately €1000, higher costs were suggested to save 831 million €.

If savings from prevented end‐of‐life cancer care were not considered at all, estimated costs per prevented YLL were €2798 under the assumptions of the main analysis and varied from €1356 to €9093 under the assumptions of the sensitivity analyses (Table 4).

**Table 4:** Estimates of costs of vitamin D supplementation per prevented year of life lost due to cancer (YLL) not considering any savings of end‐of‐life cancer care. IU, international units; YLL, years of life lost.

Scenario	Supplementation costs/person	Cancer mortality reduction^a^	Total costs per year (million €)	Prevented cancer deaths	Prevented YLL	€/prevented YLL
Base case	25€ per year	13%	900	28 842	321 671	2798
Stronger effect^b^	25€ per year	21%	900	46 591	519 623	1732
Weaker effect^c^	25€ per year	4%	900	8874	98 976	9093
400 IU per day^d^	10€ per year	11%	360	24 405	272 184	1323
2000 IU per day^e^	50€ per year	17%	1800	37 716	420 647	4279

^a^ Observed mortality reduction found in individual trials (scenario ‘400 IU per day’, ‘2000 IU per day’) or in the meta‐analysis of Keum *et al*. (all other scenarios).

^b^ Lower bound of 95% CI of effect size for cancer mortality by vitamin D supplementation (RR = 0.79) instead of 0.87.

^c^ Upper bound of 95% CI of effect size for cancer mortality by vitamin D supplementation (RR = 0.96) instead of 0.87.

^d^ Assuming that this lower daily dose of vitamin D would result in a slightly smaller cancer mortality reduction of 11%, as observed in the study by Wactawski‐Wende *et al*. ([10], see Table 2).

^e^ Assuming that this higher daily dose of vitamin D would result in a stronger cancer mortality reduction by 17%, as observed in the study by Manson *et al*. ([13], see Table 2).

## 4 Discussion

We estimated potential costs, savings of end‐of‐life cancer care, prevented cancer deaths, and YLL of daily supplementation of vitamin D in the German population aged 50 or older. Our main scenario implied prevention of approximately 29 000 cancer deaths and 322 000 YLL, costs of €900million, and savings of €1154 million, resulting in net savings of €254 million per year. The majority of sensitivity analyses also suggested vitamin D supplementation to be cost‐saving. In the most pessimistic scenario (only 4% reduction in cancer mortality), costs to save one life‐year (cost‐effectiveness ratio) were below €6000.

Even when disregarding cost savings due to prevented cancer deaths and associated end‐of‐life cancer care, vitamin D supplementation would still be highly cost‐effective: In our base case scenario, almost 322 000 life‐years could be saved at costs of €900 million, corresponding to a cost‐effectiveness ratio of €2798/YLL. In the most pessimistic scenario (only 4% cancer mortality reduction, no savings from cancer care costs, supplementation costs of €50 per year), a life‐year could be saved at costs of less than €10 000, still making vitamin D supplementation much more cost‐effective than many other therapeutic measures. Treatment costs for prostate and lung cancer in the United States amounted to more than $26 000 and $55 000, respectively, in 2007–2012. In Germany, cancer treatment costs in 2010 were estimated to amount to more than $18 000 in the last six months of life [17] and are likely to have increased markedly since then (see Table S2). A typical threshold for cost‐effectiveness of interventions is $50 000 (approximately €46 000) per quality‐adjusted life‐year, but this threshold has been criticized as being too low [18]. Novel cancer treatments are often considered cost‐effective despite much less favorable ratios [19, 20, 21, 22, 23, 24, 25] (see Table S3 for examples). Although estimates for cancer mortality reduction did not consider tumor stage, site, molecular subtypes, etc., our results suggest that daily vitamin D supplementation in the German population aged 50 years and older for reducing cancer mortality would be overall cost‐saving, or at least highly cost‐effective.

Cancer deaths can occur only among patients with cancer. In our analysis, we addressed vitamin D supplementation and its costs not only in cancer patients, but in the entire population, as it had been done in the RCTs our estimates are based upon. In those RCTs, both median or mean intervention time and median or mean follow‐up time ranged from three to seven years. One RCT [13] specifically excluded cancer patients at baseline. It seems plausible to assume that also in the other RCTs the majority of cancer deaths occurred among participants who had their cancer diagnosis after enrollment, which implies that supplementation most likely occurred partly before and partly after cancer diagnosis. It is therefore unclear to what extent supplementation within each period contributed to the reduction in cancer mortality. In case the benefit came predominantly or even exclusively from supplementation after the cancer diagnosis, targeted supplementation of cancer patients rather than population‐wide supplementation would be an even much more economic approach to reduce cancer mortality. Although direct evidence from RCTs for targeted vitamin D supplementation after cancer diagnosis is very sparse, epidemiological studies showing strongly increased mortality among cancer patients with vitamin D deficiency support suggestions that vitamin D supplementation might be particularly beneficial for this group. Vitamin D intake should not replace specific treatment options, but may help to increase treatment effectiveness by different biological mechanisms, including antioxidant capacity, anticancer stem cell effects, inhibiting growth and proliferation, and inducing apoptosis, among other mechanisms [26]. Future studies, ideally RCTs, should investigate the effects of vitamin D supplementation on cancer survival among patients with various types of cancer.

However, there may also be reasons for not restricting vitamin D supplementation to cancer patients. Higher vitamin D levels have been associated with various further favorable health effects, which we did not consider in this analysis, such as decreased systolic blood pressure [27], immune modulation [28], improved cognitive function in older adults with Alzheimer's disease [29], lower risk of acute respiratory infections, dementia, cognitive decline, and depression mainly in elderly [30], increased muscle strength [31], and positive effects on transferrin saturation and iron status [32]. Overall savings, both in terms of treatment costs and in terms of prevented YLL, might therefore go far beyond cancer‐related savings.

Although potential population‐wide vitamin D supplementation among older adults appears to be a very far‐reaching measure, it should be noted that a similarly far‐reaching measure of vitamin D supplementation has long been established at the other end of the lifespan, that is, among newborns and infants, and has helped to essentially eliminate formerly pandemic rickets in many countries. Population‐wide vitamin D supplementation through fortification of specific foods is also in place in a number of countries and has been shown to be effective in reducing prevalence of vitamin D deficiency [33].

Data on dose–response relations between vitamin D and cancer mortality are scarce. The recommended daily intake for adults in the general population is 800 IU [34]. Median daily doses in vitamin D trials ranged from 400 to 2000 IU per day in the trials, with higher doses tending to have stronger effects. For our main analysis, we chose 1000 IU per day, which would eliminate the risk of overdosing but should be sufficient and adequate for most individuals in Germany who are unaware of their vitamin D status. We also addressed doses between 400 and 2000 IU in our sensitivity analyses. Further studies are needed to determine the optimal daily intake of vitamin D.

To further reduce costs, supplementation only during the winter could be considered, because vitamin D deficiency is much less prevalent during the summer compared with winter months in Germany [35]. One could also focus on supplying those who are most likely to have vitamin D deficiency and benefit from supplements. Although much stronger effects of supplementation on cancer mortality are expected among the vitamin D deficient population [36], additional costs for vitamin D measurements would have to be considered if selective supplementation based on measured vitamin D status were to be taken into account.

Our study has several strengths. To our knowledge, it is the first study to estimate costs and cancer‐related savings of vitamin D supplementation on a national level, and the first study to estimate the impact of vitamin D supplementation on life‐years lost due to cancer mortality. The most crucial input parameter—the strength of the association (RR) between vitamin D supplementation and cancer mortality—was taken from a meta‐analysis of RCTs (evidence grade I), thus ruling out the possibility of most kinds of bias including reverse causation. We conducted sensitivity analyses regarding RR (effect size) and costs resulting from lower or higher daily doses.

Our study also has limitations. First, although evidence for the association between vitamin D supplementation and cancer mortality was taken from a meta‐analysis of RCTs, included studies were heterogeneous with respect to study populations, administered dose, dosing regimen, and length of follow‐up. However, among the five studies included in the meta‐analysis by Keum *et al*. four studies (those administering daily vitamin D doses between 400 and 2000 IU) yielded rather similar effects (RR estimates between 0.83 and 0.89). Hence, effect estimates were quite similar, even though cost estimates were different due to the different doses (Tables 3 and 4). Second, there was also uncertainty regarding the assumed cancer mortality reduction, which we addressed in further sensitivity analyses. Third, in the absence of precise data on saved cancer‐related costs from the RCTs, we based our calculations on relatively rough estimates of end‐of‐life cancer care costs. However, the results were fairly stable across a wide range of plausible values of such cost savings. Costs per saved YLL remained very low even if such savings were not considered at all. This supports the conclusion that vitamin D supplementation would be cost‐saving or at least highly cost‐effective. Fourth, the effect estimates of cancer mortality reduction were based on RCTs conducted in countries other than Germany, with the vast majority of study participants being contributed by studies from the United States. However, it appears plausible that the effects of vitamin supplementation on preventing cancer deaths might be at least as strong in Germany, where the prevalence of vitamin D deficiency after calibration according to the international Vitamin D Standardization Program (VDSP) seems to be higher (approximately 13–15%) than in the United States (approximately 6%) [35, 37, 38]. Effect estimates of vitamin D supplementation on cancer mortality were very similar in RCTs from the United States and the UK. Furthermore, the main findings were robust against variation of saved treatment costs. This supports suggestions that population‐wide supplementation of vitamin D is likely to be cost‐saving or at least highly cost‐effective across North American and Western European countries, even taking variation in cancer incidence and treatment costs into account.

In summary, our analysis suggests that population‐wide vitamin D supplementation of older adults in Germany might prevent almost 30 000 cancer deaths per year, with savings of end‐of‐life medical care for cancer patients exceeding costs for population‐wide vitamin D supplementation. Therefore, it might be an extremely effective and economic approach to limit the burden of cancer in addition to its well‐established or most likely beneficial effects on bone health and other major health outcomes. Notwithstanding the need for further studies to assess the health and economic impact of specific supplementation schemes more precisely, measures to overcome the high prevalence of hypovitaminosis D on the population level should no longer be postponed. These include, for example, food fortification or promotion of population‐wide or targeted vitamin D supplementation.

## Conflict of interest

The authors declare no conflict of interest. The funding source had no role in the study design, in the collection, analysis, and interpretation of data; in the writing of the report; and in the decision to submit the article for publication. Researchers were independent from funders. All authors had full access to all data in the study and can take responsibility for the integrity of the data and the accuracy of the data analysis.

## Author contributions

HB designed the study; TN drafted the original manuscript; TN and HB analyzed the data; TN drafted the original manuscript; and HB, BS, TG, SK, and UM critically revised the manuscript and contributed important content. TN is the guarantor of the article.

## Transparency statement

The lead author affirms that the manuscript is an honest, accurate, and transparent account of the study being reported; that no important aspects of the study have been omitted; and that any discrepancies from the study as originally planned (and, if relevant, registered) have been explained.

### Peer Review

The peer review history for this article is available at https://publons.com/publon/10.1002/1878-0261.12924.

## Supporting information


**Fig. S1.** Benefits and costs of daily vitamin D supplementation in Germany.

**Table S1.** Numbers of cancer deaths in 2016 and further life expectancy by age and sex according to life table 2016‐2018 in Germany.
**Table S2.** Examples for cancer treatment costs per patient before death.
**Table S3.** Comparison of cost‐effectiveness of other cancer prevention or treatment measures.

## Data Availability

All data used are publicly available from the sources stated in the Methods section.
