# Supplementary material for: Vitamin D supplementation to the older adult population in Germany has the cost‐saving potential of preventing almost 30 000 cancer deaths per year
Source: Mol Oncol. 2021 Mar 10;15(8):1986–94. doi: 10.1002/1878-0261.12924 (PMC8333776; doi:10.1002/1878-0261.12924)
Supplement: Supplementary file 1 — Fig. S1. Benefits and costs of daily vitamin D supplementation in Germany. [file MOL2-15-1986-s002.pptx]

## Slide 1
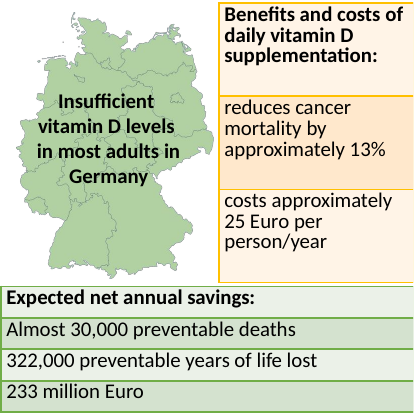

| Benefits and costs of daily vitamin D supplementation: |
| --- |
| reduces cancer mortality by approximately 13% |
| costs approximately 25 Euro per person/year |
Insufficient
vitamin D levels
in most adults in Germany
| Expected net annual savings: |
| --- |
| Almost 30,000 preventable deaths |
| 322,000 preventable years of life lost |
| 233 million Euro |
