# Supplementary material for: Vitamin D supplementation to the older adult population in Germany has the cost‐saving potential of preventing almost 30 000 cancer deaths per year
Source: Mol Oncol. 2021 Mar 10;15(8):1986–94. doi: 10.1002/1878-0261.12924 (PMC8333776; doi:10.1002/1878-0261.12924)
Supplement: Supplementary file 2 — Table S1. Numbers of cancer deaths in 2016 and further life expectancy by age and sex according to life table 2016‐2018 in Germany. Table S2. Examples for cancer treatment costs per patient before death. Table S3. Comparison of cost‐effectiveness of other cancer prevention or treatment measures. [file MOL2-15-1986-s001.docx]

**Supplementary Table 1.** Numbers of cancer deaths in 2016 and further life expectancy by age and sex according to life table 2016-2018 in Germany

|  | Men | | Women | |
| --- | --- | --- | --- | --- |
| Age | Cancer deaths | Further life expectancy^1^ | Cancer deaths | Further life expectancy^1^ |
|  |  |  |  |  |
| 50-54 | 4,698 | 27 | 4,211 | 32 |
| 55-59 | 8,266 | 22 | 6,382 | 27 |
| 60-64 | 11,839 | 17 | 8,336 | 22 |
| 65-69 | 15,032 | 12 | 10,312 | 17 |
| 70-74 | 17,730 | 10 | 12,217 | 12 |
| 75-79 | 25,324 | 9 | 18,739 | 10 |
| 80-84 | 19,240 | 7 | 16,908 | 8 |
| ≥85 | 18,744 | 4 | 23,882 | 5 |

^1^ At midpoint of the respective age interval (e.g. 52 years for age interval 50-54; for the oldest age group: at age ≥85, the median age within this group )

**Supplementary Table 2.** Examples for cancer treatment costs per patient before death

| Cancer site | Country | Year | Treatment costs in USD (time span) | Reference |
| --- | --- | --- | --- | --- |
| All (>65 years) | Germany | 2010 | 16,221 (last 6 months)  4,382 (last month) | (17) |
| All (any age) | Germany | 2010 | 18,414 (last 6 months)  4,766 (last month) | (17) |
| All | Korea | 2014 | 34,815 (total) | (36) |
| Breast | USA | 2007-2012 | 35,849 (last year) | (37) |
| Prostate | USA | 2007-2012 | 26,295 (last year) | (37) |
| Lung | USA | 2007-2012 | 55,597 (last year) | (37) |
| Colorectal | USA | 2007-2012 | 63,063 (last year) | (37) |
| All | Taiwan | 2001-2010 | Approx. 67,000 (last year)  ~30,000 (last 3 months)  ~19,000 (last month) | (38) |

Abbreviations: Approx. or ~, approximately

**Supplementary Table 3.** Comparison of cost-effectiveness of other cancer prevention or treatment measures

| Cancer site | Country | Target population | Cancer prevention or treatment measure | €/Prevented YLL or $/Prevented YLL | Reference |
| --- | --- | --- | --- | --- | --- |
| Lung cancer | Germany | Heavy former and current smokers aged 55-75 | Screening | €19,302 | (39) |
| Breast cancer | USA | Women with HR+/HER2- advanced or metastatic breast cancer | Ribociclib + Letrozole | $93,124 | (40) |
|  |  |  | Letrozole | $71,084 |  |
|  |  |  | Palbociclib + Letrozole | $102,399 |  |
|  | USA | Women with advanced triple-negative^1^ breast cancer | Chemotherapy | $61,380 | (41) |
|  |  |  | Atezolizumab | $94,965 |  |
| Colon cancer | Germany | Patients with RAS wild-type metastatic colon cancer | FOLFIRI + Cetuximab vs. FOLFIRI + Bevacizumab | €36,360 | (42) |

^1^R-negative and HER2-negative

Abbreviations: HR, hormone receptor; HER2, human epidermal growth factor receptor 2; YLL, years of life lost.
